# Fundus Autofluorescence Imaging in an Ocular Screening Program

**DOI:** 10.1155/2012/806464

**Published:** 2012-12-19

**Authors:** A. M. Kolomeyer, N. V. Nayak, B. C. Szirth, A. S. Khouri

**Affiliations:** The Institute of Ophthalmology and Visual Science, University of Medicine and Dentistry of New Jersey, Newark, NJ 07101, USA

## Abstract

*Purpose*. To describe integration of fundus autofluorescence (FAF) imaging into an ocular screening program. *Methods*. Fifty consecutive screening participants were included in this prospective pilot imaging study. Color and FAF (530/640 nm exciter/barrier filters) images were obtained with a 15.1MP Canon nonmydriatic hybrid camera. A clinician evaluated the images on site to determine need for referral. Visual acuity (VA), intraocular pressure (IOP), and ocular pathology detected by color fundus and FAF imaging modalities were recorded. *Results*. Mean ± SD age was 47.4 ± 17.3 years. Fifty-two percent were female and 58% African American. Twenty-seven percent had a comprehensive ocular examination within the past year. Mean VA was 20/39 in the right eye and 20/40 in the left eye. Mean IOP was 15 mmHg bilaterally. Positive color and/or FAF findings were identified in nine (18%) individuals with diabetic retinopathy or macular edema (*n* = 4), focal RPE defects (*n* = 2), age-related macular degeneration (*n* = 1), central serous retinopathy (*n* = 1), and ocular trauma (*n* = 1). *Conclusions*. FAF was successfully integrated in our ocular screening program and aided in the identification of ocular pathology. Larger studies examining the utility of this technology in screening programs may be warranted.

## 1. Introduction

The feasibility and effectiveness of vision screening programs for detection of glaucoma, diabetic retinopathy (DR), cataracts, age-related macular degeneration (AMD) have been described [1–3]. The majority of such programs employ digital color fundus imaging to determine whether a follow-up examination with an ophthalmologist or optometrist is required. Several investigators initiated studies examining the use of additional technologies and imaging modalities, including software-assisted digital filters, retinal thickness analyzer, and slit lamp microscopy to enhance the quality of obtained images aimed at improving detection and characterization of vision-threatening diseases (VTDs) [4–6]. In addition, using a structured questionnaire to assess patient satisfaction attending a teleophthalmology versus conventional (i.e., in-person) screening for DR, Kumari Rani et al. found that 34% of participants preferred teleophthalmology to conventional screening and 61% felt that both types of screenings were equally satisfying [7]. In this and other studies, the most common reasons given for improved patient satisfaction with teleophthalmology versus conventional screenings were cost reduction, ability to view images directly, decreased travel time, and direct consultation with a clinician [7, 8]. 

Fundus autofluorescence (FAF) is a specific wavelength light emission from lipofuscin and other molecules that accumulate in retinal pigment epithelial (RPE) cells secondary to photoreceptor oxidative damage [9]. Specific FAF patterns have been described for numerous ocular diseases and conditions, and they may also be useful in monitoring disease progression [10]. In the current study, we describe and analyze the incorporation of FAF technology in our community-based ocular screening program. 

## 2. Materials and Methods

Screening participants included homeless and working-poor individuals at soup kitchens and churches in Essex County, New Jersey. Fifty consecutive individuals were enrolled in this prospective, pilot imaging study in March 2011. This research was approved by the UMDNJ Institutional Review Board.

A detailed description of our screening program has been published previously [11]. In brief, the screening team consisted of: (1) senior medical students who completed an intake form (personal and family history of medical and ocular problems, smoking history, etc.), measured blood pressure, pulse, and oxygen saturation, assessed visual acuity (VA; SIMAV, Padova, Italy) and visual fields (frequency doubling technology; Carl Zeiss Meditec, Inc., Dublin, CA); (2) an imaging technician who measured intraocular pressure (IOP; Canon Tx-F Full Auto non-contact Tonometer, Tokyo, Japan), performed nonmydriatic color and FAF imaging (Canon CX-1 15.1 megapixel camera with CMOS sensor, Tokyo, Japan); and (3) an onsite medical director who analyzed complete screening data, reviewed color and FAF images, and made referrals for follow-up examination as needed. Those participants requiring additional follow-up examinations were referred to the eye clinic at the Institute of Ophthalmology and Visual Science at University of Medicine and Dentistry of New Jersey (Newark, NJ) that accepts uninsured patients. Presenting VA was defined as the subject's entering vision with distance correction (i.e., contacts or glasses), if applicable. Snellen VA was converted into log MAR scale, averaged, and then reconverted back into Snellen VA. By starting the screening process in a staggered fashion (i.e., at different stations), on average, 11 subjects were screened per hour (not including the time required for reviewing screening data and counseling the participants). Based on personal, social, and/or family history, information packets (available in English or Spanish) were handed out and participants were counseled accordingly. 

Nonmydriatic color and FAF images were saved and viewed on a high-resolution, wide-screen Fujitsu laptop (Kanagawa, Japan) using Digital Imaging and Communications in Medicine (DICOM)-compatible Canon Eye-Q software (Canon Medical Systems, Irvine, CA). Color images were obtained from both eyes approximately 30 seconds apart. These were evaluated side-by-side for potential changes in the optic nerve head (i.e., cup-to-disc ratio, asymmetry, neuro-retinal rim color, and nerve fiber layer integrity), blood vessels (i.e., artery-to-vein ratio and nicking, microaneurysm formation, and neovascularization), and macula and posterior pole (i.e., pigmentary changes and drusen formation). 

Following nonmydriatic color imaging, a second set of nonmydriatic images was taken in the FAF mode (530/640 nm exciter/barrier filters). Subjects were allowed to dark-adapt for 2-3 minutes. In order to prevent accommodation and pupillary constriction, both eyes were kept open while the non-imaged eye was covered. A single photograph was captured per eye at a 300 watts/second flash intensity. Two to three minutes were required in between in order for the eyes to recover. The resulting 256 monochromatic, grayscale images were then analyzed side-by-side with color images. The FAF images were analyzed in the following manner. For retinal disease (i.e., AMD, drusen), the emphasis was placed on the macular region, which was assessed for changes in fluorescence. Hypofluorescence signified RPE cell death, while hyperfluorescence suggested active expansion of area of geographic atrophy (GA). Hyperfluorescence can be classified according to specific phenotypic patterns that can be associated with AMD progression [12]. For glaucoma, the peripapillary region was of particular interest as hyperfluorescence in this area has been shown to correlate with optic nerve atrophy and/or glaucoma progression [13]. This is different from a myopic crescent, which would appear as a gray band typically spanning the temporal peripapillary region. As for DR, we focused on diabetic macular edema (DME) that may appear as areas of hyperfluorescence. 

## 3. Results

Mean ± SD age was 47.4 ± 17.3 years. Females represented 52% (*n* = 26). There were 29 (58%) African Americans, seven (14%) Caucasians, seven (14%) Hispanics, and seven (14%) others. Nine (18%) subjects had diabetes mellitus type 2 for a mean duration of 10.5 years; 13 (26%) had hypertension (based on self-reported history and/or blood pressure measurements at the screening); and 16 (32%) were active smokers. Only 14 (28%) had a dilated fundus examination within the past year. Mean VA was 20/39 in the right eye and 20/40 in the left eye. Mean ± SD IOP was 14.8 ± 3.9 mm Hg and 14.9 ± 4.4 mm Hg in the right and left eye, respectively. 

**Figure 1:**
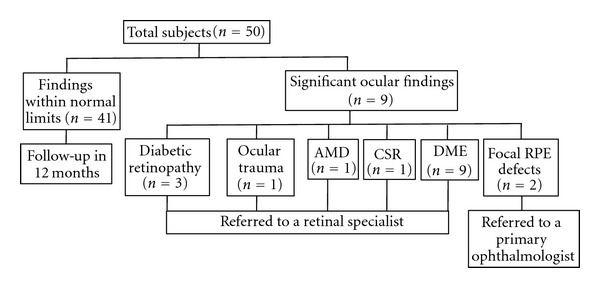
Flow chart of imaging results and referral patterns. Abbreviations: DME: diabetic macular edema; AMD: age-related macular degeneration; CSR: central serous retinopathy; RPE: retinal pigment epithelium.

**Figure 2:**
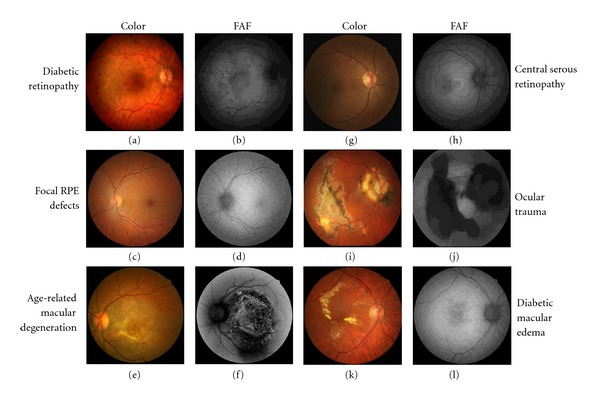
(a, b) Compared to color, FAF imaging identified a larger number of dot-blot hemorrhages and microaneurysms and was superior at outlining RPE disturbances. (c, d) Perifoveal RPE hypopigmentation was evident on the color image, while extensive focal RPE defects were seen on FAF imaging. (e, f) FAF versus color imaging showed more detail in terms of the extent of RPE disturbance. (g, h) A faint circular lesion was seen nasal to the fovea on color imaging. FAF imaging was superior to color imaging at characterizing the full extent of this lesion by delineating RPE hyperfluorescence and damage. (i, j) Extensive fibrosis was appreciated on color imaging, while FAF imaging further delineated an extensive area of RPE degeneration. (k, l) Diabetic macular edema with extensive hard exudates were clearly identified on color but not FAF imaging.

On average, 22 eyes were imaged per hour. Performing color and FAF imaging on each eye resulted in reviewing 44 images per hour. We identified nine (18%) individuals with distinct color and/or FAF patterns representing DR (*n* = 3), focal RPE defects (*n* = 2), DME (*n* = 1), AMD (*n* = 1), central serous retinopathy (CSR; *n* = 1), and ocular trauma (*n* = 1) (Figure 1). In eight (89%) of these subjects, FAF imaging improved detection and/or characterization of pathology versus color imaging. For example, in individuals with DR, FAF identified a larger number of dot-blot hemorrhages and microaneurysms, and it was superior at outlining RPE disturbance compared to color imaging (Figures 2(a) and 2(b)). While only minor perifoveal RPE hypopigmentation was evident on the color image, we identified two patients with extensive focal RPE defects after FAF imaging (Figures 2(c) and 2(d)). In the subject with AMD, FAF imaging provided more detail than color imaging in terms of the extent of RPE disturbance (Figures 2(e) and 2(f)). In an individual with chronic CSR, although we were able to appreciate a circular lesion nasal to the fovea in the color photograph, the FAF image was superior at characterizing the full extent of this lesion (e.g., RPE hyperfluorescence) (Figures 2(g) and 2(h)). In the case of ocular trauma, extensive fibrosis was appreciated on color imaging, while FAF imaging further delineated an extensive area of RPE degeneration (Figures 2(i) and 2(j)). The only pathologic finding that was better appreciated on color imaging was DME with extensive hard exudates that were not apparent on FAF imaging (Figures 2(k) and 2(l)). Based on these findings, seven (14%) subjects were referred to a retinal specialist and two (4%) to a primary ophthalmologist. All subjects were successfully imaged under the study conditions and none were referred for a dilated examination due to small pupil size.

## 4. Discussion

As far as we can discern, this is the first prospective pilot imaging study set out to determine the feasibility of integrating FAF technology in a community-based ocular screening program. We postulated that correlation of color and FAF imaging may allow for improved characterization of damage to the retina and RPE. FAF has already proved to be a useful “screening” tool in several retinal diseases/conditions, including AMD, hydroxychloroquine toxicity, and serpiginous-like choroiditis [14, 15]. Logistically during our screening, the process was aided by the fact that the Canon CX-1 camera comes specifically configured to perform color, FAF, and other types of imaging. Therefore, we did not have to be concerned about transporting an extra piece of equipment to our screenings (which commonly occur in difficult to access locations such as basements or buildings without elevators), or decreasing the number of participants screened due to space restrictions. Additionally, the inter-phased imaging software allowed for side-by-side viewing of color and FAF fundus images, which aided in comparing and correlating the ocular findings. 

An average of 22 eyes were imaged, and 44 color and FAF digital images were reviewed per hour. Identification of VTDs necessitating referral to a general or specialty ophthalmologist occurred in nine (18%) individuals. In our previous studies, an average of 26–30 eyes was imaged per hour and the rate of VTD detection was 10–30%; however, neither of these studies employed two different types of imaging modalities [11, 16]. Of the nine (18%) individuals with identified VTDs in our current study, only seven (14%) would have been referred for a comprehensive examination based on results of color fundus imaging (excluding the two participants with focal RPE defects). Thus, in addition to improved characterization of pathology identified on color fundus imaging, incorporation of FAF imaging as a complementary technology in this cohort increased ocular pathology detection in our screenings by 29% (from seven to nine eyes with pathology). 

In the present study, we were able to show successful integration of FAF imaging in a community-based ocular screening program. The imaging itself was simple to perform and it resulted in enhanced detection of abnormalities in a wide variety of retinal diseases when performed side-by-side with color fundus imaging. FAF imaging also improved our ocular pathology detection rate. Some of the potential limitations to using FAF technology in screenings include: (a) increased cost of the FAF camera versus a color fundus camera; (b) increased screening time to allow for reversal of pupil constriction secondary to higher flash intensity required to acquire FAF images; (c) our currently limited understanding of the utility of FAF in certain VTDs like glaucoma and DR; (d) requirement of patient cooperation and clear ocular media (e.g., without a visually significant cataract or vitreous opacity) in order to obtain high-quality images; and (e) potential difficulty with image interpretation due to absence of standardized equipment, protocols, and grading systems. A cost-benefit analysis, including the final impact on local health indicators, should be pursued. Although the FAF capable camera used in the present study is more expensive than a conventional color fundus camera, the potential of FAF to improve ocular disease detection warrants further study. This may be especially pertinent for retina specialists for the diagnosis, detection of progression, anticipation of prognosis, and treatment of AMD, DME, and so forth. Earlier detection may positively impact the exorbitant costs attributable to undiagnosed ocular diseases such as AMD, glaucoma, and DR [17–20]. Future work correlating FAF findings with optical coherence tomography may enhance evaluation of diseases that damage the retina, RPE, and choroid, thereby potentially avoiding false positive referrals [21]. 

Based on our findings, we believe that FAF imaging is a complementary technology that may have a significant impact on teleophthalmology applications. Advances in imaging technologies make screening for VTDs more accurate by employing complementary imaging technologies such as color fundus imaging, software-assisted analysis [16], and FAF. This may further enhance the sensitivity and specificity of referral algorithms in the future. In the current study, the number of subjects allowed for a descriptive analysis only. A larger follow-up study designed to compare the sensitivity, specificity, and positive and negative predictive values for VTD detection with and without the incorporation of FAF is warranted. More specifically, a prospective, double-armed sequential screening study in which referral patterns based on color fundus imaging only versus color fundus imaging followed by FAF imaging should be performed. 

The potential impact of improved characterization and detection of ocular pathology on disease burden (including cost to society) warrants further investigation. As a corollary, we believe that it would be pertinent to conduct cost analysis studies in order to make recommendations regarding the economic feasibility of incorporating FAF imaging in ocular screening programs. Such studies will aid in determining the full value of this technology in teleophthalmology.
